# Adapting the I-PASS Handoff Program for Emergency Department Inter-Shift Handoffs

**DOI:** 10.5811/westjem.2016.9.30574

**Published:** 2016-10-04

**Authors:** James A. Heilman, Moira Flanigan, Anna Nelson, Tom Johnson, Lalena M. Yarris

**Affiliations:** Oregon Health & Science University, Department of Emergency Medicine, Portland, Oregon

## Abstract

**Introduction:**

Academic emergency department (ED) handoffs are high-risk transfer of care events. Emergency medicine residents are inadequately trained to handle these vital transitions. We aimed to explore what modifications the I-PASS (illness severity, patient summary, action list, situation awareness and contingency plans, and synthesis by receiver) handoff system requires to be effectively modified for use in ED inter-shift handoffs.

**Methods:**

This mixed-method needs assessment conducted at an academic ED explored the suitability of the I-PASS system for ED handoffs. We conducted a literature review, focus groups, and then a survey. We sought to identify the distinctive elements of ED handoffs and discern how these could be incorporated into the I-PASS system.

**Results:**

Focus group participants agreed the patient summary should be adapted to include anticipated disposition of patient. Participants generally endorsed the order and content of the other elements of the I-PASS tool. The survey yielded several wording changes to reflect contextual differences. Themes from all qualitative sources converged to suggest changes for brevity and clarity. Most participants agreed that the I-PASS tool would be well suited to the ED setting.

**Conclusion:**

With modifications for context, brevity, and clarity, the I-PASS system may be well suited for application to the ED setting. This study provides qualitative data in support of using the I-PASS tool and concrete suggestions for how to modify the I-PASS tool for the ED. Implementation and outcome research is needed to investigate if the I-PASS tool is feasible and improves patient outcomes in the ED environment.

## INTRODUCTION

Handoffs are unique, high-risk transfer of care events. Breakdown in communication is the leading root cause of sentinel events reported to The Joint Commission (TJC).^1^ In a large multicenter study, resident physician handoffs had a baseline medical error rate of 24 errors per 100 admissions and a preventable adverse event rate of four events per 100 admissions.^2^ Due to the importance of handoffs, the Accreditation Council for Graduate Medical Education (ACGME) has built an emphasis on teaching and assessing handoff competency into its Next Accreditation System.^3^ Furthermore, the Association of American Medical Colleges (AAMC) has highlighted the importance of handoffs in medical education with the inclusion of handoffs as one of the 13 Core Entrustable Professional Activities for Entering Residency.^4^

The largest multicenter handoff study conducted to date used a bundle of interventions that included standardized education, the “I-PASS” mnemonic and an electronic handoff tool. After implementation, the study demonstrated a 26% overall reduction of medical errors in the inpatient pediatric setting.^2^ Smaller studies have shown some success in improving compliance with standardization and others have shown improvement in time of handoff or user satisfaction with the new handoff process. 5–8

Academic emergency medicine (EM) training centers present unique barriers to safe handoff processes. ED inter-shift handoffs involve coordination of care for highly complex patients under significant time constraints.^9^–^11^ Academic EM training centers require specialized educational interventions to teach and assess provider handoffs across the continuum of medical education.

We aimed to determine what modifications the I-PASS mnemonic and education bundle required to be adapted to the ED setting. We used a mixed-methods needs assessment that included literature review, focus groups and a survey. Using a conceptual framework, we sought to delineate the distinctive features of ED handoffs. We then further explored with participants these unique features in the context of the I-PASS education bundle. Finally, we attempted to obtain a consensus of modifications the I-PASS mnemonic would require to be acceptable for use in the ED setting.

## METHODS

### Settings and Participants

This mixed-methods needs assessment was conducted at an academic ED with approximately 50,000 patient visits per year. Twenty-four core faculty and 33 residents constitute the three-year EM residency program. There are also 10 adjunct emergency physicians who function as attendings in the ED. The handoff care team includes residents, attendings, charge nurses, and occasionally midlevel providers. The senior resident at each change of shift leads the handoff. The pre-existing handoff process is semi-standardized and consists of using the Situation, Background, Assessment and Recommendations (SBAR) mnemonic to organize the verbal handoff presentation. The written handoff notes are documented from the verbal presentation in the electronic medical record EPIC and do not use a standardized format. Residents, attendings, midlevel providers and charge nurses were invited to participate in the focus groups by email invitation. Only residents and attendings were invited to participate in the survey because they were most frequently involved in patient handoffs in the acute side of the ED. Midlevel providers primarily staff the ED observation unit. Participation was voluntary and confidential. The institutional review board approved this research study.

### Study Protocol

#### Literature Review Protocol

We searched PubMed and Google Scholar using the search terms “ED Handoff,” “Emergency Department Handoff,” “Handoffs,” “Inter-shift Transition of Care,” “Standardized Handoffs,” “Standardized ED Handoff,” “Implementation of Standardized Handoffs,” and “Standardization of Inter-shift Handoffs.” We identified 23 articles. Our study team reviewed the articles and created a summary of each article. All members of the research team shared comments and impressions on how the literature related to our project.

#### Focus Group Protocol

We used open-ended questions designed to investigate what participants felt were the crucial elements of ED handoffs and how these could be incorporated into the I-PASS system. Two examples of open-ended questions include the following: “If we started using this mnemonic [I-PASS] in our ED, what if anything would you recommend changing to make sure it meets our needs?” and “If a standardized sign-out process was adopted, what outcomes would you hope could be improved by implementing the process?” To a large extent, we allowed focus group discussions to proceed naturally. The facilitator participated as necessary to clarify responses and ask follow-up questions relevant to understanding the barriers and promoters of effective ED handoffs. The facilitator also directed the conversation to ensure participants addressed how key elements of the ED handoff could be incorporated into the I-PASS system. We asked participants to remember and comment on their cumulative experiences in all the EDs in which they have clinically worked. We asked about other EDs in order to increase the external validity and not be institution-specific. Due to multiple study investigators being known to the participants, a facilitator who was new to the culture and not known to the participants facilitated the focus groups. The facilitator underwent over 10 hours of training on grounded theory methodology and focus group facilitation strategies, including both independent study and mentored discussion and practice.

We used theoretical sampling strategy to recruit groups of inter-professional clinical providers who currently participate in handoffs in our ED. After collecting the initial focus group data, we continued the theoretical sampling process by integrating a midlevel provider into the focus group sessions. Early data analysis suggested that the midlevel provider perspective could lend crucial insight into the handoff phenomenon. We were able to include a midlevel provider in a subsequent focus group.

Focus group size ranged from four to eight individuals. Each focus group included individuals who had not previously participated. We conducted the focus groups in October and November 2014. Two of the four focus groups were composed of a mixed group of residents, attendings and charge nurses. One of these focus groups also included a physician assistant. The other two focus groups included only residents and attendings.

#### Survey Protocol

##### Survey Content and Administration

We conducted a literature review of previous surveys done on ED handoffs and identified one study as a model.^12^ We based the first half of the survey questions on this study. Since there were not previous studies done on adapting the I-PASS system to the ED setting, for the second half of the survey we created open-ended questions that probed participants for how this new system would be best adapted to the ED setting. The survey instrument underwent content review to improve clarity along with cognitive interviews for validation of content and response process. We conducted the survey during November and December of 2014. The survey was administered through SurveyMonkey® and participants included residents and attendings.

### Data Analysis

We used a grounded theory approach along with a constructivist/interpretivist paradigm to evaluate the perceptions of clinical providers who participate in the handoff process in the ED.^13^–^16^ We used theoretical sampling, an iterative process, and a constant comparative method of data analysis. Our primary aim was to delineate the unique features of ED handoffs and then determine if these unique features could be incorporated into the I-PASS education bundle. Finally, we attempted to develop a consensus of modifications that the I-PASS mnemonic and education would require to be acceptable for use in the ED setting.

Data analysis began with reviewing notes taken from focus group sessions and then analyzing the hand transcription of focus group audio. Participant data was de-identified. Two team members separately analyzed and coded the data using an iterative process of theme and subtheme identification. To ensure the trustworthiness and credibility of data analysis we compared focus group transcripts with observer notes, along with the hand-transcribed session notes. We used a separate process for the data from the survey. We disabled the IP address tracking to ensure that none of the responses in the SurveyMonkey^®^ survey was linked to a particular individual. Two team members analyzed and coded survey data using an iterative process of theme and subtheme identification. Team members compared the focus group and survey theme and subtheme identification by performing triangulation with the goal of obtaining a deeper understanding of the handoff process.

## RESULTS

Focus group participants suggested adapting the patient summary to include anticipated disposition of patient. If necessary, the verbal handoff should include events leading to ED presentation and ED course as part of the patient summary. Participants generally agreed that including illness severity initially was important. Additionally, participants commented that the action list helped to frame the role of the oncoming team by “[a]llow[ing] the listener to frame what their role in the patient’s care will be – to ‘watch,’ to ‘follow up labs and dispo’ or ‘start from scratch.’” Summary by receiver also had suggested modification of application to the ED handoff process. Since each patient handoff in the ED is brief, the majority of participants agreed that the summary of each patient should be included after all patients were presented. Thus, the summary provides one or two sentences for each patient as part of an overall summary of all the patients included in the ED handoff. The table summarizes the themes and subthemes identified through our focus groups and survey.

Twenty-two of 31 residents (71%) and 22 of 32 (68%) attendings responded to the survey. Two residents and two attendings were not included in the survey due to conducting this research study. The survey was analyzed independently from the focus groups, and results yielded no significant content additions to the themes and subthemes identified in the focus groups. However, the survey did yield several wording changes to reflect contextual differences.

Themes from all qualitative sources converged to suggest changes for brevity and clarity. See Figure for a summary of the modifications to the I-PASS mnemonic. At the end of each of the focus group sessions, participants were read back the suggested changes to the I-PASS tool by the facilitator. A dominant theme included *acceptance of change* (Table ) *--* most participants agreed that the I-PASS tool would be well suited to the ED setting.

## DISCUSSION

The I-PASS bundle of interventions used in the multicenter trial, in the inpatient pediatric setting, included a robust set of standardized education curriculum, job aids and formalized processes to ensure residents and faculty were adhering to the I-PASS method of handoffs.^2^ The major components included two hours of didactic presentations, one hour of simulation, a collection of job aids, faculty development resources and faculty observation tools to assess resident handoffs. The education included known best practices of communication including the TeamSTEPPS*^TM^* model. In addition to education on known best practices, there is specific education and training on the I-PASS mnemonic that was created by the study group.^17^

The purpose of our study was to explore whether the I-PASS mnemonic could be adapted to the ED setting. If a modified ED I-PASS mnemonic could be developed, then only minor modifications would be required to adapt and then pilot the original I-PASS bundle of interventions in the EM provider setting. Our qualitative findings demonstrate that the I-PASS mnemonic may be acceptable in the ED setting with certain modifications to accommodate the time constraints and dynamic nature of patient care within the ED.

We identified three major themes that influence modifications to the I-PASS handoff: time, order and culture. Multiple participants commented that the patient summary and summary by receiver required modification for use in the ED.

This mixed-methods needs assessment is the first to explore if the I-PASS handoff system could be used in the ED setting. Our literature review demonstrated that there has been limited research of ED handoff improvement bundles. Due to cost and complexity, none of these ED studies have demonstrated a reduction in medical errors due to the transition-in-care intervention. However, the I-PASS bundle of interventions has been shown to reduce medical errors during handoffs in the inpatient pediatric setting. Our research provides qualitative evidence that the I-PASS bundle of interventions could be adapted for use in the ED. Future research will be needed on the feasibility of adapting these interventions and to determine if using a modified I-PASS bundle reduces medical errors related to inter-shift handoffs in the ED setting.

## LIMITATIONS

This mixed-methods study is limited by the single center. Although we asked participants to rely on their cumulative experiences in all prior clinical settings in exploring their perceptions regarding ED handoffs, future studies assessing the impact of the I-PASS intervention in the ED setting should include multiple centers to ensure external validity. We made efforts to ensure thematic saturation and data credibility, but it is possible there are additional relevant themes that were not uncovered by our study. Although the sampling and focus group structure was designed to facilitate inter-professional discussion, additional themes may have been uncovered if groups were separated by discipline.

## CONCLUSION

A standardized handoff system may address concerns about ED inter-shift handoff safety, efficiency, and effectiveness. With modifications for context, brevity, and clarity, the I-PASS system appears well suited for application to the unique, time-sensitive ED setting. This study is important because it provides qualitative data in support of using the I-PASS tool in the ED environment and concrete suggestions for how to modify the I-PASS tool for the ED. Implementation and outcome research is needed to investigate if use of the I-PASS tool is feasible and improves patient outcomes in the ED environment.

## Figures and Tables

**Figure:**
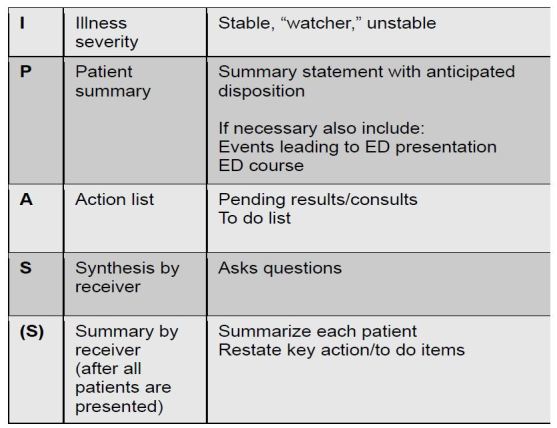
Emergency department-adapted I-PASS (illness severity, patient summary, action list, situation awareness and contingency plans, and synthesis by receiver).

**Table:** Themes, subthemes and discussion of ED adaptation of I-PASS, a mnemonic (illness severity, patient summary, action list, situation awareness and contingency plans, and synthesis by receiver) for patient handoff.

Themes and subthemes	Representative quotes	Discussion for ED adaptation
Time		
Time + order	“I think we need to do it at the end of all the patients and have it be very brief, otherwise our sign out will be too long” “A disadvantage to I-PASS would be a longer signout, due to the mnemonic as a whole or because of a specific aspect”	Summary by Receiver should wait until all the patients’ handoffs have occurred and should be very brief. Important to engage and educate residents and staff to reinforce goal of I-PASS and consider timing previous signout and comparing to I-PASS signout.
Time as environment	“I think we need a blocked out time for sign out – it is already a long process because we are constantly being interrupted by nursing staff, which throws everything off and then things get missed… maybe the signing out team goes to a separate area for signout so we aren’t interrupted”	Important to engage and include nursing staff in the handoff process in order to minimize interruptions.
Time + safety	“Need uninterrupted time in quiet space to allow for safer transition handoffs”	Important to optimize staffing and space to provide protected time for handoff.
Order		
Storytelling – how	“For patient summary, we can keep it shorter – for example, we don’t need the full hospital course, just a brief synopsis of ED care”	Shorten Patient Summary for ED setting and lead with disposition to help frame presentation.
Storytelling - content	Benefit of I-PASS is “pointed action plan rather than nebulous recommendations” “Allows the listener to frame what their role in the patient’s care will be: to ‘watch’, to ‘follow up labs and dispo’ or ‘start from scratch’”	Agreement that the I-PASS system helps to provide specific items to follow up and plan. Agreement that I-PASS system provides a useful structure to frame the oncoming team’s role in the patient’s care. Assists the team to create a shared mental model.
Culture		
Ways of thinking	“I-PASS is more aligned with ED thinking”; “[previous process] never made sense to me. I-PASS seems very similar to what I am doing now without any particular training” I-PASS as “more like real life what we need to know; less artificial”	
Ways of learning	The last two S’s in your [mnemonic] are meaningless without seeing the patient. You cannot truly know what is ‘going on’ if you have not laid eyes on it.” “Training people. Sticking to the script”; “Everyone learning it and getting acclimated”; “Forcing providers to consistently use it”; “Everyone adopting or trying to give sign out in this way to someone who doesn’t like it” “Learning a new system is usually inefficient until all users are up to speed.”	
Reticence to change	“[I-PASS is] not helpful at all… Don’t need another mnemonic”; “Don’t really like it that much”; “Don’t really like mnemonics. Would not use it”. “Dislike either [mnemonic device]. Like to just tell about the patient. Say what is important” “culture of individuality, old habits, hard to practice and implement change when you’re already tired”	
Acceptance of change	“ I like it. It seems easy and useful”; “I-PASS would need to demonstrate better utility than SBAR*” “Seems reasonable to try, as long as it doesn’t increase duration of the sign-out”	
Environment	“My concern isn’t the mnemonic, honestly. It’s everything else. (Frequent interruptions, people insisting on giving prolonged ‘one liners’ on patients who are discharged, etc.)”	
How tools are used	“I feel like [I-PASS] should have a written component though… by the passer or the receiver. With multiple patients often being handed off, its easy to cross wires with plans” “I-PASS would need to demonstrate better utility than SBAR*, but even so, may not be used properly”; or, inability to fully integrate existing tools into current culture: “I like it [I-PASS] and think you could make it work if it was incorporated into our system rather than making an extra ‘note’ or boxes that you have to fill out”	Necessary to have both a verbal and written structure and process for the I-PASS system in the ED. Success depends on education, training and reinforcement of any handoff process, especially when new residents start the year. Engage faculty with the handoff process. Incorporation of I-PASS into the existing unique culture and environment can be important for acceptance of new process.
Team Dynamics and interactions	“The last two letters however force the idea of recapping key points.” I-PASS as an advantage because it “in-corporates… closed loop communication”; “I-PASS provides clear communication”	

*SBAR,* Situation Background Assessment Recommendation.
